# Astrocyte Activation is A Potential Mechanism Underlying Depressed Mood and Apathy in People with HIV

**DOI:** 10.13188/2332-3469.1000048

**Published:** 2022-12-26

**Authors:** Ronald J. Ellis, Yan Fan, David Grelotti, Bin Tang, Scott Letendre, Johnny J. He

**Affiliations:** 1 Departments of Neurosciences and Psychiatry, University of California, San Diego, CA, United States; 2 Department of Ophthalmology, UT Southwestern Medical Center, Dallas TX, United States; 3 Department of Psychiatry, University of California, San Diego, CA, United States; 4 Departments of Medicine and Psychiatry, University of California, San Diego, CA, United States; 5 Department of Microbiology and Immunology, Chicago Medical School Rosalind Franklin University, North Chicago, IL, United States

**Keywords:** HIV, Depression, Astrocyte

## Abstract

**Background:**

Astrocytes become activated with certain infections, and this might alter the brain to trigger or worsen depressed mood. Indeed, astrocytes are chronically activated in people with HIV infection (PWH), who are much more frequently depressed than people without HIV (PWoH). A particularly disabling component of depression in PWH is apathy, a loss of interest, motivation, emotion, and goal-directed behavior. We tested the hypothesis that depression and apathy in PWH would be associated with higher levels of a biomarker of astrocyte activation, glial fibrillary acidic protein (GFAP), in cerebrospinal fluid (CSF).

**Methods:**

We evaluated PWH in a prospective observational study using the Beck Depression Inventory-II (BDI-II) and additional standardized assessments, including lumbar puncture. We measured GFAP in CSF with a customized direct sandwich ELISA method. Data were analyzed using ANOVA and multivariable regression.

**Results:**

Participants were 212 PWH, mean (SD) age 40.9±9.14 years, median (IQR) nadir and current CD4 199 (57, 326) and 411 (259, 579), 65.1% on ART, 67.3% virally suppressed. Higher CSF GFAP correlated with worse total BDI-II total scores (Pearson correlation r=0.158, p-value=0.0211), and with worse apathy scores (r=0.205, p=0.0027). The correlation between apathy/depression and GFAP was not in fluenced by other factors such as age or HIV suppression status.

**Conclusions:**

Astrocyte activation, reflected in higher levels of CSF GFAP, was associated with worse depression and apathy in PWH. Interventions to reduce astrocyte activation -- for example, using a peptide-1 receptor (GLP-1R) agonist -- might be studied to evaluate their impact on disabling depression in PWH.

## Introduction

Despite viral suppression on combination antiretroviral therapy (ART), people with HIV (PWH) suffer from a higher prevalence of depression than the general population. Depression is the most common psychiatric comorbidity in HIV [1–3] and apathy – a lack of interest, motivation, emotion, and goal-directed behavior – is a particularly prominent and frequent manifestation of depression in PWH [4]. While often related to depression, apathy also occurs in other brain disorders where dopaminergic neurotransmission is disrupted, such as abulia and akinetic mutism. Dopaminergic dysfunction is also common in PWH [5]. PWH who have depression and apathy show poorer medication adherence [6], lower rates of viral suppression [7,8] poorer quality of life [9,10], and shorter survival [11–14]. Chronic systemic and neuro-inflammation persist in virally suppressed PWH and predict morbidity and mortality [15]. Apathy and anhedonia are linked to inflammation [16] as evidenced by elevated levels of interleukin-(IL-)6 and tumor necrosis factor (TNF)-α [17–22]. Inflammation, anhedonia, and apathy often signal resistance to traditional antidepressants [23–29]. In the brain, activated astrocytes mediate many aspects of immune function and inflammation. Astrocyte activation is an important contributor to neuronal-glial network dysfunction in depression [30,31], as would be expected based on the central role of astrocytes in brain metabolism and inflammatory signaling. Astrocyte activation at autopsy is associated with antemortem depressed mood [32,33]. Expression of glial fibrillary acidic protein (GFAP) is upregulated in activated astrocytes [34], and cerebrospinal fluid (CSF) levels of GFAP, a marker of astrocyte activation, are increased in people with depression [35]. Astrocyte activation is a prominent feature of brain disease in HIV and correlateswith the release of neurotoxicviral proteins such as Tat [36–41]. Although apathy is a particularly prominent and disabling component of depression in PWH and HIV causes astrocyte activation, no previous study has evaluated CSF GFAP levels in PWH in the setting of depression and apathy. We tested the hypothesis that elevated CSF GFAP levels, reflecting astrocyte activation, would be correlated with depressed mood and apathy in PWH.

## Methods

This cross-sectional study evaluated PWH at 6 US centers in CNS AntiRetroviral Effects Research (CHARTER), a prospective longitudinal cohort, between 2003–2008. Inclusion criteria were HIV seropositivity and ability to complete the protocol. Participants who had severe neuropsychiatric comorbidities (e.g., untreated schizophrenia or seizure disorder) were excluded. All study procedures were approved by local Institutional Review Boards and all participants provided written informed consent for the study procedures, including future use of data and biospecimens.

All participants were comprehensively evaluated with standardized assessments including lumbar puncture, phlebotomy, neuromedical history and examination, and laboratory testing. A trained clinical examiner interviewed and examined participants to collect information such as antiretroviral treatments, nadir CD4+ T cell counts, and history of diabetes mellitus.

Depressive symptoms were assessed using the Beck Depression Inventory (BDI-II), a validated survey of 21 questions that assess depressive symptoms and their severity [42]. Higher BDI values indicate higher severity depressive symptoms with a value >13 indicating at least mild depression. The BDI-II includes three standard subscales capturing cognitive, somatic, and affective symptoms of depression. Since we predicted that the apathy component of depressed mood would be particularly important in HIV, we constructed an apathy subscale using items that specifically address apathy symptoms: loss of pleasure, loss of interest, indecisiveness, and tiredness or fatigue (range 0–5, higher scores indicate worse atrophy).Dependence in instrumental activities of daily living (IADLs) was assessed using a modified version of the Lawton and Brody Scale [43] that asks participants to rate their current and best lifetime levels of independence for 13 major IADLs such as shopping, financial management, transportation, and medication management [9]. Individuals who reported difficulties in completing >2 IADLs were considered functionally dependent.

### Clinical Laboratory Evaluations

HIV infection was diagnosed using an enzyme-linked immunosorbent assay with Western blot confirmation. HIV RNA in plasma was measured using commercial assays and deemed undetectable at a lower limit of quantification (LLQ) of 50 copies/mL. CD4+ T lymphocytes were measured by flow cytometry and nadir CD4+ T lymphocyte count was assessed by self-report.

CSF GFAP in picograms per milliliter (pg/mL)was measured by a customized direct sandwich ELISA method, with a mouse monoclonal antibody cocktail against GFAP (Covance, Cat#SM1–26R) as the capturing antibody and a rabbit polyclonal anti-GFAP antibody (DAKO, Cat# Z0334) as the detection antibody. GFAP protein standards (Calbiochem, Cat# 345996) were used to standardize concentration curves.

### Statistical Analyses

Demographic and clinical characteristics were summarized using means and standard deviations, medians, and interquartile ranges or percentages, as appropriate. Log_10_ transformation was used to normalize CSF GFAP values. The Pearson correlation coefficient was used to measure the relationship of GFAPlevels to indices of depressed mood and apathy. We applied ANOVA when the distribution of the outcome variable was not significantly different from normal. When distributions significantly deviated from normal, non-parametric analyses were conducted. Follow-up analyses used recursive partitioning to identify informative GFAP cut-offs. We adjusted for testing multiple related outcomes using the Benjamini Hochberg procedure. When potential statistically confounding variables such as age and demographic and disease variables were significantly related to both the predictor (CSF GFAP level) and outcomes of interest (apathy, depression), we evaluated these further in multivariable regression analyses. Relevant covariates considered included demographics, HIV disease and treatment parameters, and antidepressant treatments. Analyses were conducted using JMP Pro version 15.0.0 (SAS Institute, Cary, NC, 2018).

## Results

The sample included 212 participants with a mean±SD age 40.9±9.14 years, female17.9%, black40.6%, non-Hispanic white 47.6%,Hispanic 8.96%, other race/ethnicity 2.83%, non-Hispanic white 47.6%, median (IQR) duration of HIV infection 7.3 (2.58, 12.8) years, current CD4 411 (259, 579), nadir CD4199 (57, 326), plasma HIV RNA suppressed (<50 copies/mL) in32.7%, CSF HIV RNA suppressed in62.3%. The mean±SD log_10_ CSF GFAP level was 3.47±0.0781pg/mL, and the mean BDI-II score was 12.2±9.84, with 39.2% having a BDI-II>13, reflecting at least mild depression.

### Potential statistical confounds

#### Demographics

Several variables were significantly related to depression parameters and GFAP levels. Older individuals had both worse apathy (r=0.220, p=0.0013) and higher log_10_CSF GFAP (r=0.357, p=9.00e-8). In a multivariable regression predicting apathy scores, the interaction between age and GFAP was not significant(p=0.888), while both main effects of both GFAP (p=0.0442) and age (p=0.0197) were significant. Apathy scores were not related to sex or ethnicity. Older PWH also had worse overall depressed mood (BDI-II total score; r=0.155, p=0.0305). In a multivariable regression predicting BDI-II total score, the main effect of age (p=0.164) and the interaction of age with GFAP (p=0.846) were not significant.

Sex and ethnicity were not significantly related toBDI-II total score. However, both sex and ethnicity were related to CSF GFAP. Males had higher GFAP levels than females (mean±SD 3.48±0.0793 versus 3.44±0.0635, p=0.0033) and whites had higher levels than the other ethnicities (non-Hispanic white [3.49±0.081] versus [black, 3.46±0.076] versus Hispanic [3.44±0.060] versus other race/ethnicities [3.450.031]; p=0.0051). In a multivariable regression predicting BDI-II total score, GFAP was statistically significant (p=0.0165), while sex and the interaction term were not(ps>0.25).In a similar regression for ethnicity, GFAP was significant (0.0239), while ethnicity(p=0.237) and the interaction (p=0.891) were not.

#### Antidepressant medications

The proportion of participants taking antidepressant medications was 32%. The odds of taking antidepressants for those with a BDI-II>13 was 2.50 (95% confidence interval 1.38, 4.54). Antidepressant use was associated with worse apathy scores (3.45±2.34 versus 1.94±2.11, p=5.43e-6) and higher GFAP levels (3.46±0.0789 versus 3.50±0.0706, p=0.0003).PWH both on and off antidepressant medications contributed to the relationship between GFAP and apathy: for those on at least one antidepressant: r=0.123, p=0.295, N=67; for those not on any antidepressants: r=0.139, p=0.0988, N=142;interaction p=0.895.

#### Laboratory Parameters

PWH with detectable plasma viral loads had worse total BDI-II scores (13.2±9.93 versus10.2±9.43, p=0.0409)as well as worse scores on the cognitive (4.82±4.76 versus 2.94±3.86, p=0.0049), but not apathy, somatic or affective items (ps=0.0990, 0.3893 and 0.0856, respectively). Both virally suppressed and unsuppressed PWH contributed to the relationship between GFAP and apathy: for suppressed PWH r=0.126, p=0.302, N=69; for not suppressed r=0.262, p=0.0016, N=142; interaction p=0.388. Detectable viral load was not associated with CSF GFAP(3.47±0.0797 versus 3.48±0.0748, p=0.160). In a multivariable regression predicting BDI-II cognitive scores, the main effects of both GFAP (p=0.00139) and detectable viral load were significant (p=0.00316), but their interaction was not (p=0.438). Findings were similar for the BDI-II total score (data not shown). Those with suppressed CSF HIV RNA had better apathy scores (2.17±2.26 versus 2.85±2.29, p=0.0347). Higher GFAP correlated with higher CSF total protein (r=0.310, p=4.51e-6), but CSF protein did not relate to apathy scores (r=0.092, p=0.183). GFAP was not influenced by CSF leukocyte count (r=−0.0136, p=0.845). Apathy scores did not correlate with current (r=0.0353, p=0.610) or nadir CD4+ T cells (r=0.00936, p=0.893), or plasma viral suppression (suppressed 2.058±2.60 versus unsuppressed 2.61±2.99, p=0.0990).

In a multivariable regression predicting BDI-II, GFAP was significant while being on an antidepressant and its interaction with GFAP were not (ps=0.153 and 0.359). In a stepwise multivariable regression (p to enter 0.05, p to leave 0.05) predicting BDI-II total score from CSF GFAP, age, sex, ethnicity, nadir, and current CD4+ T-cell count and viral suppression, the model selected CSF GFAP (p=0.00915) and lack of viral suppression(p=0.00998) as the best correlates (overall model p=0.0046).

#### Impact of Depression on Activities of Daily Living and Quality of Life:

PWH with worse depression (higher BDI-II scores) had worse HIV-MOS physical health summary scores (r=−0.626, p=1.85e-23), and worse mental health summary scores (r=−0.825, p=1.25e-51). Similarly, higher CSF GFAP correlated with worse physical (r=−0.177, p=0.0116)and mental (−0.196, 0.0052) health scores. The proportion of participants reporting dependence in instrumental activities of daily living (IADLs) was 15.6%; participants with a BDI-II>13 had 11.9-fold higher odds of being dependent (95% CI 4.68, 36.8; p=1.57e-8). There was a 3% increase in the odds of having detectable plasma viral load per one-unit increase in BDI-II scores increased (OR 1.03 [95% CI 1.00, 1.07] per 1-point increase in BDI-II,p=0.0372). Similarly, the odds of having detectable CSF viral load increased as BDI-II scores increased (OR 1.03 [1.01, 1.06] per 1-unit increase in BDI-II, p=0.0194).

## Discussion

This is the first study to show that PWH with worse apathy and other attributes of depressed mood had higher levels of GFAP in CSF. Since in the central nervous system GFAP is found only in astrocytes, and since its expression is upregulated in activated astrocytes [34], higher CSF GFAP concentrations are believed to reflect greater astrocyte activation. Astrocytes are known to be activated in HIV infection [36–41] and to influence brain circuits involved in mood and motivation [30,31,35]. This study’s principal finding that depression in PWH was associated with higher CSF GFAP levels was robust to consideration of a variety of important demographic and disease-related potential confounds. Our data are consistent with previous research on the role of astrocyte activation in depression in PWoH [30,31] and extend these findings to PWH. Consistent with the existing literature [44], worse depressed mood in this study was associated with several adverse outcomes including poorer viral suppression and independence in instrumental activities of daily living, highlighting the clinical impact of depressed mood in PWH.

We suggest that the impact of astrocyte activation on depression is via neurotoxicity [37]. Astrocytes, among other functions, are responsible for metabolic support to neurons [45,46] and are involved in neuronal repair [47]; activation of astrocytes diverts their resources from neuronal support. Astrocyte activation related to HIV infection may confer greater vulnerability to depression in PWH, a biological risk factor that may explain the higher prevalence of depression in PWH.

Strengths of this work include the careful characterization of depressed mood and the consideration of a range of potential confounding factors, to which the primary findings were robust. The cohort was multicenter and racially diverse, enhancing generalizability. Limitations of this study include its cross-sectional design, limiting causal inference. Based on existing knowledge, a causal link between astrocyte activation, as indexed by CSF GFAP, and depressed mood is plausible; however, it is conceivable that changes in activity, diet and other lifestyle factors associated with depression might lead to astrocyte activation (reverse causation). Statistical confounds were not detected in this study; however, an unmeasured variable might account for the association between GFAP and depressed mood. The effect sizes demonstrated here were small, albeit statistically significant. Females were underrepresented. The rate of viral suppression was lower than in many modern cohorts; however, after adjustment for viral suppression, elevated CSF GFAP levels were still significantly associated with depressed mood. Antidepressant medications could have been taken for reasons other than depressed mood, such as for neuropathic pain.

These findings raise the possibility of interventions, potentially influencing pathways that might affect depressed mood [48–51]. For example, using a glucagon-like peptide-1 receptor (GLP-1R) agonist [52,53] or the synthetic cannabinoid R(+)WIN 55,212–2, both of which inhibit astrocyte activation [31,48]. A future clinical trial may fruitfully explore this therapeutic option for depressed PWH, particularly those who fail to respond to traditional antidepressant treatments.

## Figures and Tables

**Figure 1:**
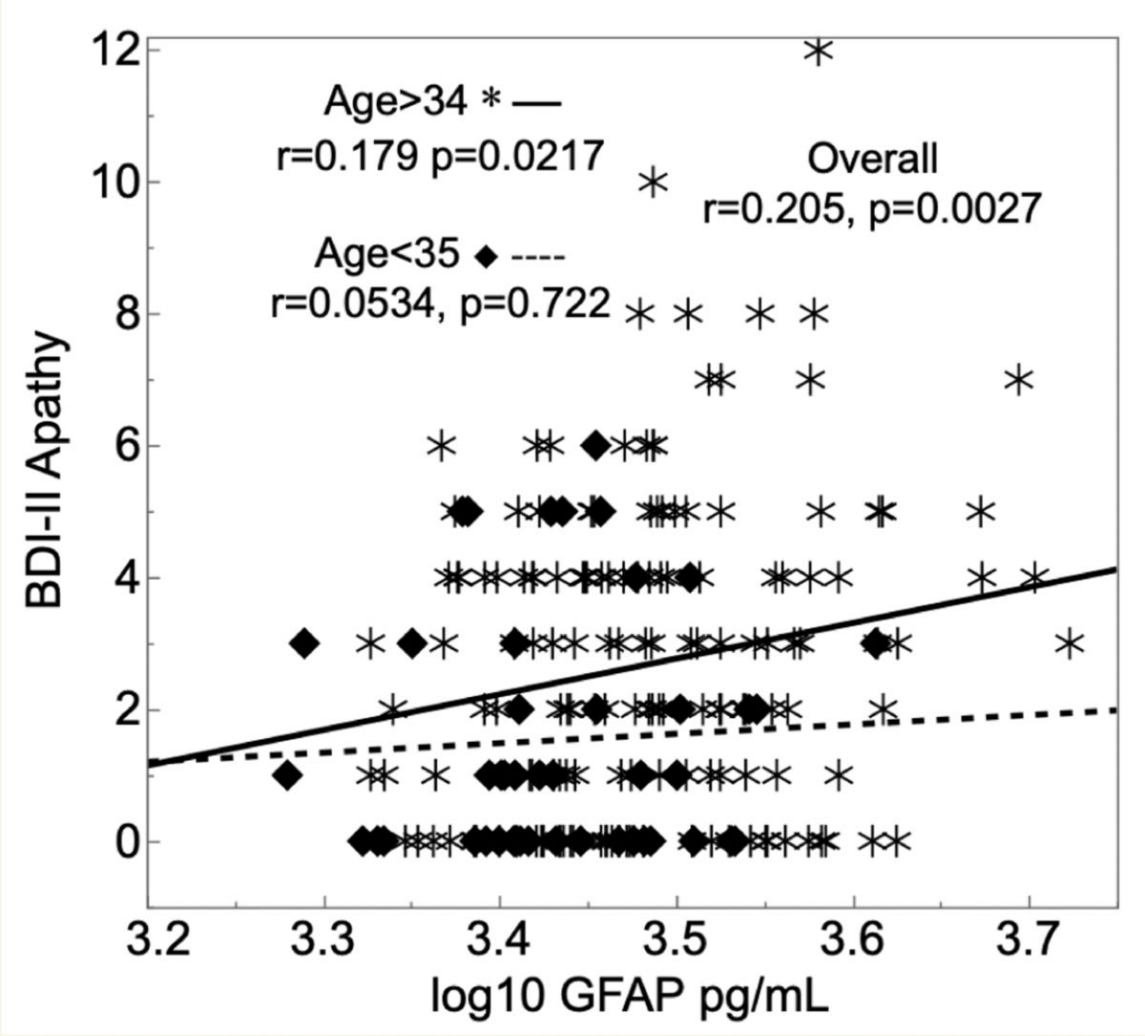
PWH with higher levels of glial fibrillary acidic protein (GFAP) in cerebrospinal fluid (CSF; x-axis) had worse BDI-II apathy subscale scores (y-axis). This relationship was significant for those older than 34 years (asterisks), but not for younger participants (diamonds).

**Table 1:** Higher CSF GFAP levels (greater astrocyte activation) correlated with worse Beck Depression Inventory-II (BDI-II) total and subscale scores (higher = worse depression).

	*Pearson correlation (r)*	* p-value*
Total BDI-IIscore	0.158	0.0211
BDI-II subscales		
Cognitive	0.135	0.0494
Apathy*	0.205	0.0027
Somatic	0.144	0.0359
Affective	0.154	0.0252

* Apathy symptoms: loss of pleasure, loss of interest, indecisiveness, and tiredness or fatigue (range 0–5, higher scores indicate worse atrophy) Table 1 shows Pearson correlations between log_10_CSF GFAP and BDI-II and each of its subscales
